# Immunology and Oxidative Stress in Multiple Sclerosis: Clinical and Basic Approach

**DOI:** 10.1155/2013/708659

**Published:** 2013-09-24

**Authors:** Genaro G. Ortiz, Fermín P. Pacheco-Moisés, Oscar K. Bitzer-Quintero, Ana C. Ramírez-Anguiano, Luis J. Flores-Alvarado, Viridiana Ramírez-Ramírez, Miguel A. Macias-Islas, Erandis D. Torres-Sánchez

**Affiliations:** ^1^Laboratorio de Mitocondria-Estrés Oxidativo y Patología, División de Neurociencias, Centro de Investigación Biomédica de Occidente del Instituto Mexicano del Seguro Social, Sierra Mojada 800, CP 44340 Guadalajara, Jalisco, Mexico; ^2^Departamento de Química, Centro Universitario de Ciencias de Ciencias Exactas e Ingenierías, Universidad de Guadalajara, Blvd. Marcelino García Barragán 1421 CP 44430 Guadalajara, Jalisco, Mexico; ^3^Laboratorio de Neuroinmunomodulación, División de Neurociencias, Centro de Investigación Biomédica de Occidente del Instituto Mexicano del Seguro Social, Sierra Mojada 800, CP 44340 Guadalajara, Jalisco, Mexico; ^4^Departamento de Bioquímica, Centro Universitario de Ciencias de Ciencias Exactas de la Salud, Universidad de Guadalajara, Sierra Mojada 950 CP 44350 Guadalajara, Jalisco, Mexico; ^5^Departamento de Neurología, Unidad Médica de Alta Especialidad, Centro Médico Nacional de Occidente del Instituto Mexicano del Seguro Social, Belisario Dominguez 1000 CP 44340 Guadalajara, Jalisco, Mexico

## Abstract

Multiple sclerosis (MS) exhibits many of the hallmarks of an inflammatory autoimmune disorder including breakdown of the blood-brain barrier (BBB), the recruitment of lymphocytes, microglia, and macrophages to lesion sites, the presence of multiple lesions, generally being more pronounced in the brain stem and spinal cord, the predominantly perivascular location of lesions, the temporal maturation of lesions from inflammation through demyelination, to gliosis and partial remyelination, and the presence of immunoglobulin in the central nervous system and cerebrospinal fluid. Lymphocytes activated in the periphery infiltrate the central nervous system to trigger a local immune response that ultimately damages myelin and axons. Pro-inflammatory cytokines amplify the inflammatory cascade by compromising the BBB, recruiting immune cells from the periphery, and activating resident microglia. inflammation-associated oxidative burst in activated microglia and macrophages plays an important role in the demyelination and free radical-mediated tissue injury in the pathogenesis of MS. The inflammatory environment in demyelinating lesions leads to the generation of oxygen- and nitrogen-free radicals as well as proinflammatory cytokines which contribute to the development and progression of the disease. Inflammation can lead to oxidative stress and vice versa. Thus, oxidative stress and inflammation are involved in a self-perpetuating cycle.

## 1. Introduction 

Multiple sclerosis is a chronic inflammatory demyelinating disease of the central nervous system and a common cause of disability in young adults. The loss of myelin results in a multitude of neurological impairments. Myelin is composed of a lipid bilayer membrane that encircles itself around axons, which is critical for neural signaling and transmission. Myelin insulates the axon from potentially harmful exogenous factors while enhancing neural impulse transmission efficacy from one part of the CNS to another. Myelin membrane and its producing cell (oligodendrocyte) are destroyed in MS. 

Typically, the disease affects the brain, spinal cord, and optic nerves in the CNS and spares the nerve roots and peripheral nerves in the peripheral nervous system. The interplay between inflammatory and neurodegenerative processes in MS typically results in intermittent neurological disturbance (episodes of acute worsening) followed by progressive accumulation of disability. During an MS attack, inflammation occurs in areas of the white matter of CNS in patches called “plaques.” This process is followed by the destruction of myelin in the brain and spinal cord, leading to diminished or lost function. A wide range of clinical symptoms include motor dysfunction, fatigue, tremor, nystagmus, acute paralysis, loss of coordination or balance, numbness, disturbances in speech and vision, and cognitive impairment. Usually MS begins as a relapsing-remitting process and evolves into a secondary progressive stage with accumulating disability. Most people experience their first symptoms of MS between the ages of 20 and 40, but there have been documented cases in young children and elderly adults. The clinical heterogeneity of MS, as well as the finding of different pathological patterns, suggests that MS may be a spectrum of diseases that may represent different processes. This large diversity of symptoms and their variability confound both the diagnosis and understanding of MS [1–5]. 

The role of genetics and environmental factors in MS is complex. Factors such as geographical location, ethnic background, and clustering in temperate climates all contribute to susceptibility. Individuals with a North European heritage are statistically more susceptible to MS than those from a more tropical environment, and it is more common in women. Genetic studies indicate that although MHC genes clearly contribute to disease susceptibility and/or resistance, it is probable that a combination of environmental factors may additionally contribute to disease development. It develops in genetically susceptible individuals after exposure to environmental factors such as vitamin D deficiency and Epstein-Barr viral infection. Multiple sclerosis may have effects that extend beyond loss of the myelin sheath. Some axons are destroyed, probably as a result of inflammatory processes in the overlying myelin and/or loss of trophic support of the axon by oligodendrocytes [6, 7].

Multiple etiologies including autoimmunity, infectious agents, environmental triggers, and hereditary factors have been proposed. However, there is substantial evidence to indicate that dysregulated immune responses, including immune mechanisms directed against myelin proteins, have a role in triggering disease onset [1, 2, 8]. 

## 2. Multiple Sclerosis Trait 

Multiple sclerosis trait is a systemic nonpathological condition not involving the nervous system parenchyma that may affect some persons who are genetically susceptible to MS. It results from the action of an antigenic challenge on the immune system of a genetically vulnerable person, that does not cause damage to the nervous parenchyma; it may never evolve into MS disease. A subsequent environmental viral-antigenic event in some people can change the trait into the disease. The increased immune response that underlies the pathogenesis of MS is the response to various antigenic challenges, either from infections, most likely viral, or possibly also from some viral vaccinations [9]. 

This trait is characterized by an exaggerated response to a number of viral antigens; the presence of oligoclonal bands in the cerebrospinal fluid; an increased vulnerability of the BBB; and being clinically and radiologically silent. Therefore, its time of activation is impossible to recognize. The pathogenic mechanism of MS is initiated by an inflammatory phenomenon that results in a much more severe alteration of the BBB. When people with the trait enter a different environment, new antigens are encountered (viruses or vaccine components), among which are some that exhibit molecular mimicry with the original trait immune activator and are also compatible with one of the person's immune receptors. 

The critical age for acquisition of MS is around 15 years. MS is almost twice more common in women, who have a greater immune reactivity and stronger reactions against infections and immunizations, than in men because of the marked effect of sex hormones. Furthermore, women have higher levels of circulating immunoglobulins and a more frequent production of a variety of autoreactive antibodies. Sex hormones have been shown to modulate a large variety of mechanisms involved in the immune response, including cell trafficking, cytokine production, lymphocyte proliferation, expression of adhesion molecules, and HLA-class II receptors. Estrogens have stimulating effects on B-cell functions, which seem to be dependent on the inhibition of suppressor T-cell events preceding the clinical onset of MS which include head and other traumas, tonsillectomy and appendectomy, allergies, and ether anesthesia. Others were infections and animal contacts. Epigenetic profiles may represent a link between environmental factors and phenotypic differences [2, 8, 10]. 

## 3. Immune Function in Multiple Sclerosis 

Because of the altered immune function in patients with MS, some questions arise, for example: (1) what is the nature of immune dysfunction?; (2) what functional defects in myelin contribute to the disease?; (3) are there any changes in development and maturation of central nervous system myelin in MS patients?; (4) what extrinsic factors triggers an immune response?; (5) what causes axonal damage and neuronal death?; (6) what novel treatments for multiple sclerosis are available? [1, 11]. It is likely that the immune system presents a pathological demyelination generated by the loss of tolerance to myelin. It under apoptosis of CD8(+) suppressor myelin-specific and the presence of immunocompetent cells that exhibited a destructive pattern to the appearance of new immunocompetent cells against myelin or by-products generated by the intraplaque macrophage activity. Activated by two essential interleukins for this purpose: interleukin 12 and interleukin 23 (as we shall see below), there is an immune response against antigens, included within the healthy central nervous system [12, 13]. Confirmation of this uncontrolled immune response based on the cooperation of two types of lymphocytes (B and T) and their relationship with neurons resulted in the cerebrospinal fluid by increasing immunoglobulins; as a result, that gives the increase in the kinetics of synthesis of immunoglobulin G, its index intrathecal, and the presence or oligoclonal distribution (oligoclonal pattern) of IgG. The presence of free light chains (kappa or lambda) and the mixed reaction MRZ2 (polyspecific and chronic) are additional parameters that contribute to this understanding of the pathogenesis [3, 14]. 

At the cellular level, there is a slight pleocytosis showed by flow cytometry and the persistence of a higher percentage of B cells which act to amplify the immune reaction and generate intrathecal IgG, IgA, and IgM [15]. Axonal transection and loss in early lesions are recent, and there is even apoptosis of cortical neurons, apparently for lack of trophic factors. The initial immune-mediated and inflammatory mechanism is followed by a neurodegenerative phenomenon that clearly dominates in time, and it could be seen by the absence of enhancement on MRI and immunomodulation failure [16–18]. In this sense, the multiple sclerosis destructive process is an autoimmunity mixture of immune dysregulation (regulatory failure or loss of tolerance) initiated by an infectious agent of the environment (by molecular mimicry). Typically, MS begins as an inflammatory process but later develops a neurodegenerative component, which may progress independently of inflammation. An alternative view is that MS is a neurodegenerative process that is exacerbated by secondary inflammation that provokes demyelination. Thus, the rational treatment of multiple sclerosis should be directed towards all pathophysiological mechanisms and essentially be multifaceted and multidirectional [19, 20]. 

Axonal demyelination makes the individual more vulnerable to environmental stressor agents. It has been shown that acute lesions begin with phagocytosis of normal myelin sheaths by macrophages (MQs) in the presence of infiltrating T cells [20–22]. It has been demonstrated in studies with animal models of MS, the experimental autoimmune encephalomyelitis (EAE), where the primary event is the infiltration of lymphocytes and autoreactive CD4+ T cells that destroy myelin sheaths [23]. However, in some cases stress or death of oligodendrocytes could be an initial event in the MS presentation [24, 25]. The death of oligodendrocytes and the formation of new lesions may be triggered by a viral infection, followed by microglial activation and the recruitment of CD4, CD8 T cells, and macrophages (MQs) within the lesions [26]. 

T cells, particularly CD4, are considered as the initiators and primary drivers of disease. The evidence supporting this hypothesis shows that the main genetic contribution to multiple sclerosis susceptibility resides in the gene region of the major histocompatibility complex class II (MHC-II) which plays a key role in the development of the central tolerance of the T cells [27, 28]. 

Focal inflammatory demyelinating lesions are characterized by perivascular infiltrates containing predominantly clonally expanded CD8+ T cells (Tc), CD4 (Th) T cells, *γδ* T cells, monocytes, and some B cells and plasma cells. These active lesions contain a significant number of macrophages with myelin debris in their interiors as well as a significant deposit of complement factors and immunoglobulins [29]; while progressive inflammation is not observed at the beginning of relapsing-remitting multiple sclerosis (RRMS), progressing to a more advanced disease is characterized by the gradual expansion of the lesions with the presence of macrophages with myelin debris in the distal area of the inflammatory plaques, diffused abnormal inflammation, and neuronal and axonal loss in the spine associated with the degree of meningeal inflammation [29]. Potential mechanisms of induction of degenerative progression include damage of Wallerian secondary degeneration demyelination and axonal transection by reactive oxygen species and nitric oxide, in addition to energy failure by mitochondrial dysfunction [30]. An alternative to this hypothesis suggests that the progressive degeneration can also be caused by alterations in the compartmentalization of the BBB, coupled with a severe inflammatory response [31]. Although it has been shown that the early RRMS is mediated by the adaptive immune system, with waves of T cells entering to the CNS from the peripheral immune system, it is also possible that inflammation of the CNS in patients with secondary progressive multiple sclerosis (SPMS) is mediated by the innate immune response. SPMS has a slowly expanding demyelinating lesion with only modest inflammatory cell cuffing and axonal damage is also considered to be a major cause of secondary progression with irreversible neurological impairment. The main effectors to this process are microglia/macrophages and their toxic products [32]. Over the past decade, there has been an important identified variety of defects related to the autotolerance. Dysfunction in the central tolerance leads to the exit of the thymus of potent T cells presenting alterations in the T-cells receptor (TCR), which favors the appearance of autoimmune reactions; in addition, the premature involution of thymus results in a reduced export of naive T-regulatory (Treg) cells, a clone of suppressor T cells. Recent data have demonstrated the key role of the Tregs in suppressing responses of Th1 and Th17 effector cells, and this immunosuppressive activity is altered in patients with MS. These observations suggest that a defect in the autotolerance homeostasis might be the primary event of the onset of MS, which in turn induces the subsequent immune attack, inflammation, and neurodegeneration [33–35]. 

The concept that MS is a primarily neuroimmune disease is controversial. One of the characteristics that define an autoimmune disease is the failure of the immune system to keep the autotolerance against tissue-specific antigens (TSAs). MS shares physiopathological mechanisms with other autoimmune disorders, as well as an important genetic predisposition and epigenetic and environmental factors [21]. It has been suggested that interactions between genetics, epigenetics, and environmental risk factors can determine the phenotype of a neuroimmune disorder [21, 36]. 

Central tolerance could be a direct result of the elimination, in thymus, of autoreactive T cells and of the bone marrow of autoreactive B cells, which leads to a generation of T cells and mature B cells that recognize exogenous pathogens and are able to deploy auto-tolerance to autoantigens [33, 37]. Positive selection test of the ability of TCR for signaling in response to MHC class II autopeptides was deployed by cortical thymic epithelial cells (cTECs). Thymocytes expressing TCRs with few or no affinity for the MHC-II complex are sent to apoptosis. 

Surviving thymocytes pass to the thymus marrow area where bone marrow thymic endothelial cells (mTECs) express TSAs. Negative selection removes thymocytes, via active apoptosis, through a strong signal in response to MHC autopeptides; this negative selection is primarily mediated by the gene of the autoimmune regulator which encodes for the Aire protein, which in turn modulates the expression of TSAs. Recently, Aire protein has been identified in peripheral lymphoid organs which regulates the expression of TSAs, different from those expressed in the thymus [33, 34]. 

Protection against autoimmunity in a specific organ requires a minimum expression threshold relative to the TSA. Thymocytes that receive signals just after the threshold of negative selection may enter into a program of differentiation toward natural regulatory T cells (nTreg), CD4+ CD25+ Foxp3+. It is important to highlight that the mTECs and dendritic cells (DCs) contribute in the process of negative selection of naive T cells and the generation of nTregs [33, 38, 39]. The presence of autoreactive T cells in the periphery of MS patients and healthy controls shows that central tolerance is not completely efficient, and the escape of negative selection may be due to a dysfunction in the TCR or a weak bond of the autopeptide to MHC-II, which destabilizes the TCR complex. Alterations in the crystallographic structure of TCR have been identified in MS patients. In these patients the T-cell receptor and peptide-MHC complex showed alterations in its docking mode; the TCR was not aligned in the central portion of the MBP-MHC complex, but it was aligned to the N-terminal portion; consequently the stability of the entire complex of TCR-MBP-HMC was markedly reduced, its affinity was suboptimal, and self-reacting thymocytes avoided negative selection [40]. 

Another important aspect of thymic dysfunction in patients with MS is its inability to maintain the T-cell homeostasis. Patients with RRMS showed a global defect in T-cell naive output; these patients have levels below normal of the newly exported naïve T cells (TREC), suggesting premature involution of the thymus in MS [38]. TREC levels decrease to normal with age in healthy controls, while there is no association between TREC decrease and increase of age in patients with MS [35, 41]. Peripheral tolerance is regulated by complex mechanisms intrinsic to T cells, like costimulation pathways, transcriptional mechanisms, epigenetic, and other extrinsic mechanisms such as the Treg cells [42, 43]. 

In addition to the TCR-peptide-MHC complex, costimulatory molecules are also necessary to activate T cells. In the absence of these signals, T cells become anergic and induce autotolerance. Cluster of Differentiation 28 (CD28) is one of the molecules expressed on T cells that provide costimulatory signals, which are required for T-cell activation. CD28 is the receptor for CD80 (B7.1) and CD86 (B7.2). When activated by Toll-like receptor ligands, the CD80 expression is upregulated in antigen presenting cells (APCs). Stimulation through CD28, in addition to the T-cell receptor (TCR), can provide a potent costimulatory signal to T cells for the production of various interleukins (IL-6 in particular). CD4 CD28 null T cells have been found in peripheral blood of patients with MS. They are less sensitive to the regulatory mechanisms and produce high levels of IFN*γ*. Inducible costimulator protein (ICOS) has recently been identified as a new member of the CD28 family of T-cell costimulatory molecules; it binds to B7H and modulates the production of Th1/Th2 cytokines. Interestingly, RRMS patients have a lower expression of ICOS gene than in healthy controls. CD40 molecule plays a dual role in MS pathology: binding of CD40 by its ligand (CD40L = CD154) in mTECs can contribute to the development of central tolerance, and CD40 promotes differentiation of naive CD4+ T cells towards Th17. In brain lesions of patients with MS, CD40L is expressed in CD4+ cells activated in SPMS, but not in RRMS, and this higher expression in peripheral lymphocytes could be associated with the change of RR to SPMS. Cytotoxic T-lymphocyte antigen-4 (CTLA-4) binds to its receptor similar to CD28, but with a negative regulatory function. The expression of CTLA-4 is low both in SP and RRMS, but the small percentage of cells that express CTLA-4 could be associated with the primary transition of RR to SPMS. An important molecule is programmed death pathway-1 (PD-1); it is expressed by activated T cells. In MS lesions there has been a significant overregulation of its ligand, PD-L1. This molecule has been identified as an important downregulator of T-cell response in MS. 4-1BB (CD137) expression is downregulated in Tregs and DCs in patients with MS. The soluble form of the ligand (s4-1BBL) is very high in plasma and cerebrospinal fluid (CSF) in MS patients [44–47]. CD58 mRNA expression is significantly increased in patients with MS; its protective effect occurs in part by an increase in the expression of Foxp3 transcriptional factor in Treg cells [48]. In relation to the transcriptional mechanisms, it has been demonstrated that Foxp3 is the transcription factor responsible for the differentiation of CD4 and CD8 cells towards T-regulatory cells (Tregs) CD4+ CD25+. Foxp3 represents a heterogeneous population that lacks a specific marker of Treg cell phenotype. Besides CD25, Foxp3 is a master regulator gene in these cells and is fundamental in the maintenance of homeostasis and autotolerance. Foxp3 protein expression at the cellular level correlates with the suppressor activity of Treg cells. The natural-Treg cells (nTregs) are antigens functionally mature and primed in the thymus before meeting antigens in the periphery to prevent autoimmunity. They migrate to peripheral lymphoid organs. Within, lymph nodes interact with DCs and block their ability to prime the CD4 naive cells and to bring them to their subsequent differentiation to autoreactive and autospecific T cells; T cells exert their activity by cell-cell contact [39]. Foxp3 is able to send the CD4 naive cells into an inducible regulatory T cells (iTreg) phenotype, which is functionally similar to the nTreg but differs in the epigenetic regulation of Foxp3, its effect does not require cell-cell contact, and it is primarily mediated by cytokines such as IL-10, IL-35, and TGF-*β*. The differentiation of nTregs from iTregs can be rescheduled by IL-6 through the lineage of Th17 cells [49]. Tregs number consistently increased in CSF of patients with MS compared with healthy controls, but a defect was detected in its suppressor function in RRMS patients associated with a very low expression of Foxp3 mRNA; this is not presented in SPMS patients, where the Tregs suppressor function and Foxp3 expression are normal [28, 49]. 

A subtype of Tregs carries markers, like CD39 and CD73, which are involved in the regulation of the suppression of toxicity induced by IL-17. CD39 and CD73 are potent immunosuppressive molecules. Patients with RRMS exhibit a marked reduction in the number of CD39-circulating molecules [28, 49]. The Tregs express low levels of CD127 (IL-17 receptor *α* chain), a marker of activation that is negatively correlated with the expression of Foxp3 and its suppressive activity. When the CD4+ CD25+ CD127 low Tregs are segregated into naive T cells and memory T cells (Tmem), activity of naive Tregs is reduced after costimulation with CD3 in RR and SPMS patients [50]. 

Other types of T cells involved in the pathogenesis of MS and EAE include the Tr1 cells. T cells are characterized by secreting large amounts of IL-10. In patients with MS, a marked defect in the induction of Tr1 cells has been observed. Th3 cells are regulatory T cells producing TGF*β* generated in patients after oral ingestion of myelin antigens. The HLA-G represents a subtype of T-regulatory cells, CD25 and Foxp3, which exert their suppressor activity independently of IL-10 and IFN-*β*. Clinical correlation between levels of sHLA-G in CSF and the severity of symptoms in MS has been reported [49, 50]. 

Treg cells CD8+ CD25+ Foxp3+, as well as CD4, are a heterogeneous population. They can be induced by T cells CD8+ CD25− on a continuous antigenic stimulation. CD8-Tregs cells specifically recognize and lyse activated myelin-specific T cells; this cytotoxic response is decreased in peripheral blood and CSF of MS patients during episodes of disease exacerbation. Historically, MS has been considered as an autoimmune disease mediated mainly by CD4 T cells, and associated, at least in part, with alleles, MHC-II. However, it has also been described as having a significant association with MHC-I molecules. In addition CD8 T-cell frequency is much higher than that of CD4 cells in inflammatory plates, and CD8 shows an oligoclonal expansion in plates, CSF, and circulation. The CD8 T cells express CD45RA, and the chemokine receptor CCR7 is in the peripheral blood. Possible phenotypes for these cells include CD45RA+ CCR7+; another subtype, CD45RA− CCR7+, that represents cells with antigenic experience but without effector functions enabled has been defined as central memory T cells (TCM); a subtype of memory T cells which CD45RA− CCR7−, shares effector functions, and known as effector memory T cells (Tem); finally a subtype CD45RA+ CCR7− are the most differentiated type of memory cells. 

There is an association between HLA-A0301 of the MHC-I allele and susceptibility to MS, and it has been suggested that MHC-I is restricted to the CD8 T cells in the pathogenesis of MS, but otherwise, the allele HLA-A0201 of the MHC-I confers significant protection against disease [28, 49–51]. 

Most of the resident cells in CNS, such as astrocytes, oligodendrocytes, and neurons, express MHC-I, at least in inflammatory conditions. All these cells become potential “targets” for the CD8 T cells. The upregulation of MHC-I molecules can be observed during the early stages in the course of the disease, after a demyelination episode. In the postmortem material tissue of patients with RRMSCD8 T cells are detected in MS lesions, preferentially in the parenchyma and in greater amount than their CD4 counterparts. Furthermore, CD8 T lymphocytes with polarized cytolytic granules are observed in close apposition to oligodendrocytes and demyelinated axons, which suggests a positive correlation between the extent of axon damage and the numbers of CD8 T cellls.

A recent report describes that the CD8 T cells producing IL-17 (Tc17) are involved in the MS pathogenesis. This report is based on the observation that the vast majority of the CD8 T cells detected in vascular spaces of active MS lesions produce IL-17, in contrast to the inactive lesions, where a small amount of IL-17-producing T cells are present [28, 49–51]. The T cells expressing *γδ* phenotype provide the first line of defense against infections, especially on mucous membranes, through the secretion of IFN-*γ*. The *γδ* T cells directly recognize ligands induced by stress, inflammation, and infection. That latter phenomenon includes nonpeptidic antigens, alkylamines, phosphoantigens, MHC-I nonclassic molecules, like MIC-A and MIC-B, and heat shock proteins. The *γδ* T cells act as a source of cytokine in innate immunity and exhibit a potential influence on adaptive immune responses. Expanded clones of *γδ* T cells in acute brain lesions of MS and in CSF of patients are found at the onset of the disease, suggesting that the *γδ* T cells do contribute to the neuroinflammatory process. In contrast, in few models of EAE, a protective role of *γδ* T cells was observed [4]. During infectious processes the *γδ* T cells, as well as the *αβ* T cells, are able to produce IL-17, which opens up the possibility that this IL-17 derived from *γδ* T cells could contribute to the pathophysiology of MS and EAE in CNS [11]. 

Homeostasis and function of the B cells in the CNS are relevant in the development and clinical course of MS defined as “clinically definite” (CDMS). In the 85% of patients with MS, the initial presentation is defined as a clinically isolated syndrome (CIS); approximately 60% of the cases of CIS develop a second demyelinating event and are diagnosed as CDMS in the course of the next 20 years. CSF B-cell count is increased in patients with CIS, and B-cell subtypes showed a marked expression of the *α*4 subunit of VLA-4 receptor, necessary for their migration through the BBB. The abnormal activity of the B cells not only is present in the first manifestations of disease, but also is present in its conversion to CDMS [3]. 

Ectopic lymphoid follicle structures have been described in the meninges of CDMS patients. These structures contain proliferative B cells, suggesting germinal centers presence surrounding the brain. Moreover, few immune patrolling cells such as T cells and DCs have been identified in the CSF and meninges of those CDMS patients. The subpial cortex close to the meningeal follicles shows a high gradient of neuronal loss and axonal degeneration, which suggests that there might be cytotoxic elements partially responsible for cortical demyelination in the gray matter pathology [52]. The presence of markers and B cell activation correlates with the activity and progression of CDMS; CXCL13 levels are increased in CIS, in RRMS, and in SPMS, and this correlates positively with the total count of B cells in CSF, with the intrathecal synthesis of IgG, and with the appearance of markers for demyelination [3]. Inflammatory B-cell aggregates or germinal centers observed in the subarachnoid spaces in cases of SPMS are indicative factors of disease severity [14]. 

## 4. Cytokines 

MS is characterized by perivascular inflammation and high levels of circulating T and B lymphocytes that assault to myelin protein, thereby suggesting a role for immunoregulatory cytokines [53]. Interleukin (IL) 1*β*, tumor necrosis factor alpha (TNF*α*), and IL-6 are cytokines which are increased during immune activation and inflammation [54]. In MS, TNF*α* plays a role in the demyelination phenomenon, IL-1*β* might be responsible for T-cell activation, and IL-6 participates in the immunoglobulin (Ig) synthesis in the central nervous system. The neuroinflammatory processes of vascular cuffing, destruction of the blood-brain barrier, reactivation of autoreactive lymphocytes, demyelination, and neuronal toxicity have been studied in patients in the last three decades; inflammation is based on histopathological observations and the presence of cytokines in cerebrospinal fluid and in serum. 

Increased TNF*α* level precedes clinical manifestation and is associated with the expanded disability status scale (EDSS) [55–57]. Additionally, MS is associated with the parallel upregulation of other proinflammatory cytokines such as interferon-*γ* (INF-*γ*), lymphotoxin-*α*, IL-2, IL-6, IL-12, IL-17, IL-23, IL-33, and downregulation antiinflammatory cytokines such as transforming growth factor-*β* (TGF-*β*), IL-4, and IL-10. TGF-*β* plays an important role in the regulatory T-cell synthesis; that inhibits the autoimmune response and protects against inflammatory injury [58–62]. IL-17 decreases in the progressive phase and could be considered to evaluate the immunomodulatory therapy [63].

Cytokine levels have been useful to determine the grade of inflammation and correlate with the progression of the disease.  Table 1 presents the cytokine levels in serum and CSF. Many studies have led to understanding more about MS pathology; because of this since the early 1990s, disease-modifying drugs have been introduced for the MS treatment. The standard treatment includes interferon-*β* (INF-*β*) and glatiramer acetate (GA); these are immunomodulator agents. INF-*β* inhibits proinflammatory cytokines (TNF*α*, IL-6, IL-1*β*, and IL-17) and T-cell proliferation and clinically reduces the annual rate of relapsing [64, 65]. 

**Figure 1:**
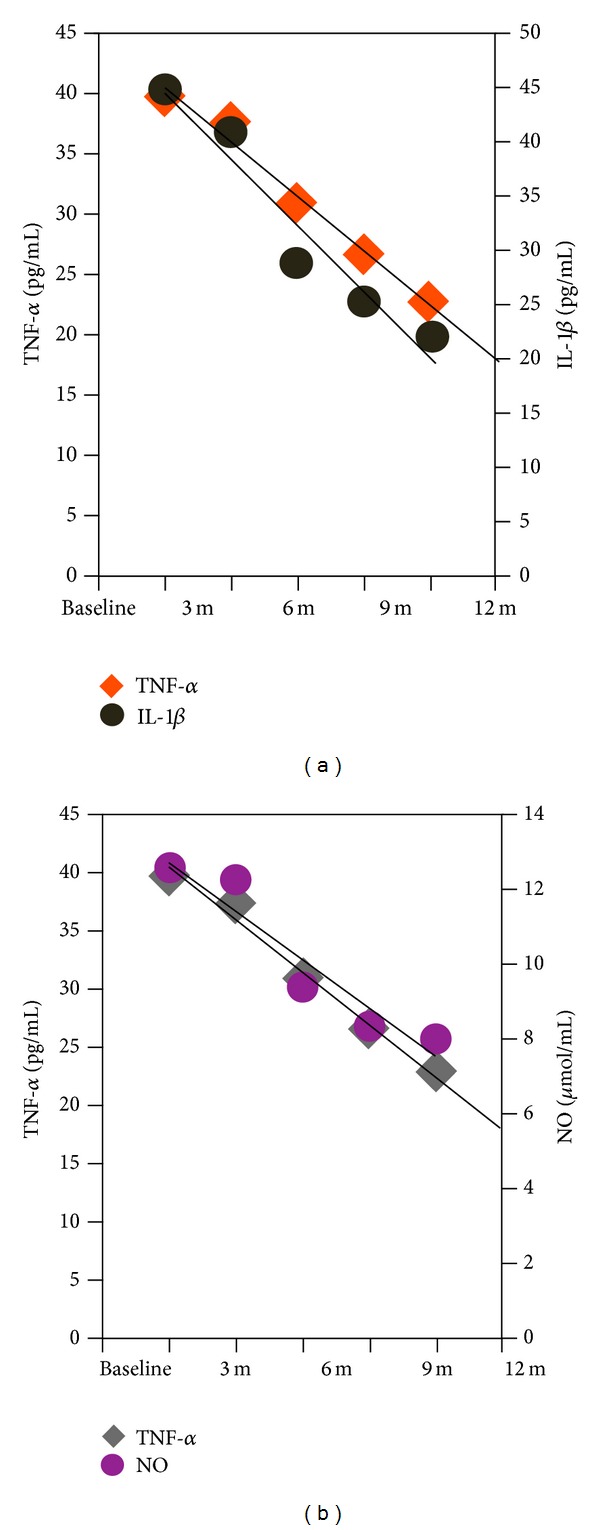
Cytokines and oxidative stress marker reduction in multiple sclerosis patients. (a) Correlation plot between TNF*α* and IL-1*β* serum levels. (b) Correlation plot between TNF*α* and nitric oxide (NO) catabolites. Pearson correlation coefficients were calculated for all data sets: IL-6 *R* ^2^ = 0.9585, TNF*α* *R* ^2^ = 0.9844, IL-1*β* *R* ^2^ = 0.9391, NO *R* ^2^ = 0.9068. Modified from Rodríguez-Rodríguez V. (2013) PhD, thesis, University of Guadalajara.

It is commonly accepted that nutrition is one of the environmental factors involved in the pathogenesis of MS.  Table 1 shows the therapeutic strategies for multiple sclerosis. The relationship between dietary omega-3 polyunsaturated fatty acids (PUFAs) intake and progression of MS remains unclear. Therefore, we developed a clinical trial in Mexican patients with relapsing-remitting multiple sclerosis. Patients were randomly assigned to either 4 g/day fish oil orally or a placebo during 12 months. Fasting blood samples were taken at 0, 3, 6, 9, and 12 months to measure TNF*α*, IL-1*β*, IL-6, and nitric oxide catabolite levels. A significant decrease was seen in serums TNF*α*, IL-1*β*, and IL-6 on catabolite levels at 3, 6, 9, and 12 months for patients in the fish oil group (see Figure 1). Therefore, 4 g of fish oil seems to have an efficacy to decrease inflammatory cytokines and nitric oxide catabolites in relapsing-remitting multiple sclerosis. 

Peripheral venous blood (10 mL) was collected from MS patients at the indicated time, into sampling tubes without EDTA. Blood was centrifuged at 3500 rpm for 5 minutes separating the serum from the cellular residue. TNF*α*, IL1b were measured using kits from R&D Systems, TNF*α* DTA00C (range of detection: 15.6–1000 pg/mL), IL-1b DLB50 (range of detection: 3.9–250 pg/mL), and NO KGE001 (range of detection: 3.12–200 *μ*mol/L).

## 5. Role of Oxidative Stress in MS 

Free radicals (pro-oxidants) are highly reactive, unstable molecules that have an unpaired electron in their outer shell and react with several cellular molecules. Reactive oxygen species (ROS) are both radical and nonradical, but reactive species derived from oxygen and physiological concentrations of ROS are a part of cell-signaling mechanisms, but much higher local concentrations are produced by immune cells in order to kill pathogens [73]. ROS are produced primarily by mitochondria as unavoidable by-products of normal cell metabolism during conversion of molecular oxygen (O_2_). The main by-products are superoxide (O_2_^−•^) and hydroperoxyl radical (HO_2_^−•^) [74]. H_2_O_2_ is formed as a by-product of the O_2_^−•^ detoxification by superoxide dismutase (SOD). The largest portion of H_2_O_2_ is converted into H_2_O and O_2_ by catalase, but some of it may escape into the cell when the H_2_O_2_-metabolising capacity of catalase is insufficient. Through Fenton reaction, H_2_O_2_ will then be converted to O_2_^−•^, the most reactive oxygen radical. Under certain conditions, formation of O_2_^−•^ will be perpetuated through the nonenzymatic Haber-Weiss reaction, requiring free intracellular iron and O_2_^−•^ or ascorbate [75].

Free radicals are also produced by cyclooxygenases, the enzymes that catalyze the rate-limiting step in the biosynthesis of prostaglandins, prostacyclins, and thromboxane A2, from their precursor arachidonic-acid [76]. Another source of oxidative stress associated with arachidonic-acid signaling in the CNS is the lipoxygenase (LOX) pathway which catalyzes the conversion of polyunsaturated fatty acids into conjugated hydroperoxides. Therefore, neuroinflammation can trigger oxidative stress by at least two different mechanisms: production of high levels of ROS by activated glia such as microglia and astrocytes and arachidonic-acid signaling through the activation of cyclooxygenase and lipoxygenase pathways [77]. On the other hand, cells have developed a defense system against oxidative reactions by generation of small molecules that can scavenge radicals, and thereby prevent cellular damage; among these are uric acid, *α*-tocopherol, ascorbate, carotenoids, and glutathione. Furthermore, cells also have enzymes such as catalase, superoxide dismutase, glutathione peroxidase, and glutathione S-transferase to enhance the efficiency of the process [68]. Oxidative stress occurs when the generation of oxidants exceeds the antioxidant capacity. Interestingly, expression of antioxidant enzymes is under the control of the transcription factor nuclear factor-E2-related factor (Nrf2), which upon oxidative stress translocates to the nucleus, where it activates antioxidant response element (ARE) mediated gene transcription [69]. Nrf2-ARE-driven genes include superoxide dismutase 1 and 2 [78], glutathione peroxidase [79], NAD(P)H:quinone oxidoreductase 1 [80], and heme oxygenase 1 [81]. Although representing only 2% of body mass, brain uses 20% of glucose and oxygen supplied to body. Postmitotic cells, such as neurons, accumulate oxidative-damaged products through the entire life. Brain is very susceptible to oxidative stress because of its high consumption of oxygen in metabolic pathways, abundant content of more easily peroxidizable fatty acids such as arachidonic acid, docosapentaenoic acid, and other omega-3 fatty acids, and low activity and quantity of antioxidant enzymes compared to other tissues. At this regard, brain is low in catalase activity containing about 10% of liver catalase. Additionally, human brain has higher levels of iron (Fe) in certain regions and in general has high levels of ascorbate. Thus, if tissue organizational disruption occurs, the Fe/ascorbate mixture is expected to be an abnormally potent pro-oxidant for brain membranes [82]. 

Oxidative stress is commonly implicated in the development of brain damage, and ROS contribute to several mechanisms underlying the pathogenesis of multiple sclerosis (MS) lesions [83]. It has been reported that ROS are produced upon interaction of monocytes with brain endothelium, which leads to tight-junction alterations, cytoskeleton rearrangements, loss of blood-brain barrier integrity, and subsequent extravasation of leukocytes into the CNS [83, 84]. Furthermore, infiltrated leukocytes produce higher amounts of ROS, which induce myelin phagocytosis and breakdown by macrophages [85], oligodendroglial damage [86], and neuronal and axonal injury [87]. Additionally, CSF levels of end products of nitric oxide metabolism are correlated with relapses, suggesting that nitric oxide plays a role in inflammatory blood-brain barrier dysfunction [88]. 

The inflammatory environment in demyelinating lesions may lead to the generation of oxygen and nitrogen free radicals as well as proinflammatory cytokines that in turn exacerbates the inflammatory response. It is clear that during oxidative burst, activated macrophages produce elevated levels of ROS and RNS by upregulation of NADPH oxidase and inducible nitric oxide synthase (iNOS), respectively, producing O_2_^−•^ and NO radicals [89]. Together, O_2_^−•^ and NO form the very harmful peroxynitrite (ONOO^•^). Peroxynitrite decays to yield oxidizing and nitrating species that react in complex ways with different relevant biomolecules. These reactive species not only induce lipid peroxidation and affect DNA or polysaccharide structure, but they also react with cellular proteins by tyrosine nitration [90]. Interestingly, iNOS is up-regulated in MS lesions [91] and in the cerebrospinal fluid of patients with MS [92]. 

## 6. Lipid Peroxidation and Protein Modification 

Free radicals can cause oxidative modification of lipids and initiate a process called lipid peroxidation (LPO). Polyunsaturated fatty acids (PUFAs) are most easily oxidized due to the easily broken carbon-double bonds. Since lipid peroxidation is a chain reaction, it can, if not rapidly inhibited, easily destroy lipid-rich areas such as cell membranes or myelin sheaths. In addition to the destruction of lipid layers, cleavage of the fatty acid carbon chains of the lipid hydroperoxides, in the presence of reduced metals or ascorbate, results in the generation of highly reactive aldehydes such as malondialdehyde (MDA), acrolein 4-hydroxy-2-nonenal (HNE), and 4-hydroxy-2-hexenal (4-HHE) [93]. These have, compared to ROS, a relatively long half-life (HNE up to 2 minutes in tissue) and can therefore diffuse to sites distant from the initial oxidative event, and they react to other molecules such as proteins or DNA [94]. HNE is a 9-carbon molecule with three reactive sites generated by cleavage of omega-6 PUFAs and mainly reacts with the amino acids cysteine, histidine, and lysine. Myelin, which surrounds the axons and is the target of the immune attacks in MS, consists of 30% protein and 70% lipids. 

Because free radical peroxidation alters the structure of biological membranes, and thereby affects their physical and chemical properties such as permeability and resorption or potential, it can be expected to play an important role in the pathomechanism of MS [95]. 

4-HNE and nitrotyrosine are present in high amounts in foamy macrophages and large hypertrophic astrocytes throughout active demyelinating MS lesions. 4-HNE can diffuse and is highly toxic to CNS cells, including oligodendrocyte precursor cells [9] and cerebral endothelial cells [96]. Interestingly, it has been shown that 4-HNE and other ROS induce tight-junction alterations, cytoskeleton rearrangements, loss of blood-brain barrier integrity, and extravasation of leucocytes into the CNS, thereby affecting endothelial cell permeability [97]. Furthermore, the presence of abundant 4-HNE-positive macrophages surrounding blood vessels suggests that enhanced perivascular 4-HNE staining may contribute to impaired blood–brain barrier functioning [98]. 

Cholesteryl ester hydroperoxides, the most abundant lipid hydroperoxides *in vivo*, derive from the oxidation of cholesteryl esters localized in the core of lipoproteins and represent a useful marker of lipid peroxidation of lipoproteins [99]. A significant increase in the plasma levels of cholesteryl ester hydroperoxides has been observed in MS patients with respect to controls [100]. Additionally, Newcombe et al. demonstrated the presence of oxidized low-density lipoprotein and lipid peroxidative end products in early and actively demyelinating plaques in postmortem MS brains [101]. Bizzozero et al. found increased amounts of protein carbonyls in the brain white and gray matter of patients with MS [102]. Van Horssen et al. showed the presence of extensive oxidative damage to proteins, lipids, and nucleotides [98, 103]. Studies performed in the CSF have shown increased levels of lipid peroxidation markers such as pentane and ethane [104], malondialdehyde (MDA) [92], and isoprostanes [105], while advanced glycoxidation end-product levels have been found normal. Nitrotyrosine, a marker for peroxynitrite formation, was detected in active demyelinated MS lesions [106]. 

## 7. Antioxidants in MS 

Antioxidant enzymes, such as superoxide dismutase 1 and 2, catalase, and heme oxygenase 1, are markedly upregulated in active demyelinating MS lesions compared to normal appearing white matter and white matter tissue from non-neurological control brains. In particular, hypertrophic astrocytes and myelin-laden macrophages expressed an array of antioxidant enzymes [98]. Enhanced antioxidant enzyme production in inflammatory MS lesions may reflect an adaptive defense mechanism to reduce ROS-induced cellular damage. 

Paraoxonase is an enzyme able to hydrolyse preformed oxidized lipids, and it exerts a protective role against oxidative damage of cells and lipoproteins. Paraoxonase activity is significantly lower in the plasma of patients affected by MS with respect to healthy subjects [66]. Previous studies have reported that levels of uric acid were increased while glutathione and *α*-tocopherol were decreased in plaques from patients with multiple sclerosis. In addition, *α*-tocopherol levels were increased in distant white matter, and normal ascorbic acid, cysteine, tyrosine, and tryptophan levels were found in plaques and in distant white matter of MS patients when compared with controls [98, 103, 107]. Van Horssen et al. reported a marked upregulation of the antioxidant enzymes superoxide-dismutases 1 and 2, catalase, heme oxygenase 1, and NAD(P)-quinone oxidoreductase 1 in active demyelinating MS lesions [108] in MS patients when compared with healthy controls. The activity of glutathione reductase has been found increased in MS patients [92]. The role of glutathione peroxidase activity in peripheral tissues of MS patients remains unclear; for example, it has been reported as a decreased enzymatic activity in blood cells [109], increased activity [110], or a normal activity in lymphocytes, granulocytes, and platelets of MS patients [111]. 

Glutathione peroxidase is a free-radical scavenger enzyme, and therefore it is an important part of the antioxidant cellular defense system. The increase in its activity found in the serum of MS subjects could be a response to oxidative stress in order to minimize cell injury. It has been reported that in the demyelinating plaques, there were decreased glutathione and *α*-tocopherol concentrations, normal ascorbic acids, and increased uric acid levels [71]. It has been reported that cerebrospinal fluid *α*-tocopherol levels and serum coenzyme Q10 levels were normal [112, 113]. Kastenbauer et al. [114] reported normal CSF levels of uric acid and its oxidation product allantoin, while [115] Amorini et al. reported increased CSF levels of uric acid and its precursor's hypoxanthine and xanthine. Lower serum uric acid level in MS patients has been reported [116]. 

Serum levels of nitric oxide metabolites (nitrates/nitrites) and lipid peroxidation products (malondialdehyde plus 4-hydroxy alkenals) were significantly increased in subjects with relapsing-remitting multiple sclerosis in comparison with those of healthy controls [67]. The higher levels of oxide nitric metabolites could be relevant in MS development if there is a low concentration of serum uric acid. Recent evidence suggests that nitric oxide contributes to the disease process of MS by blood-brain barrier breakdown, by direct tissue damage, blocking axonal conduction, and by inducing axonal degeneration and plaque formation [117]. In addition, peroxynitrite mediates several potentially destructive chemical reactions, including tyrosine nitration and lipid peroxidation, and its effects are consistently associated with active lesions during the remitting phase. Peroxynitrite participates in neuron and oligodendrocyte damage in association with inflammatory processes [118]. In addition, lipid peroxidation can trigger the process of apoptosis, activating the intrinsic suicide pathway present within all cells [119]. 

It is believed that the inflammatory environment in demyelinating lesions leads to the generation of oxygen- and nitrogen-free radicals as well as proinflammatory cytokines which contribute to the development and progression of the disease [120]. Inflammation can lead to oxidative stress and vice versa. Thus, oxidative stress and inflammation are involved in a self-perpetuating cycle. 

## 8. Role of NF**κ**B 

NF*κ*B is a DNA-binding transcriptional factor complex that interacts with promoter areas in proinflammatory genes. Oxidative stress causes an activation of the transcriptional factor NF*κ*B, which in turn upregulates proinflammatory gene expression. Strong inducers of NF*κ*B activation are the cytokines tumor necrosis factor alpha (TNF-*α*) and interleukin-1*β* (IL-1*β*). Most of the identified stimuli for NF*κ*B activation induce oxidative stress in cells [70]. Conversely, the overexpression of glutathione peroxidase inhibits the cytokine-induced activation of NF*κ*B [72]. Furthermore, the TNF-*α* induced NF*κ*B activation is attenuated by overexpression of *γ*-glutamylcysteine synthase, the rate-limiting enzyme for GSH synthesis [121]. Activation of NF*κ*B promotes transcription of proinflammatory genes that include cell adhesion molecules, such as intercellular adhesion molecule-1 and VCAM-1, enzymes, such as iNOS and cyclooxygenase-2 (COX-2), cytokines, such as IL-1*β*, interleukin-6 (IL-6), and TNF-*α*, and chemokines, such as regulated upon-normal T-cell expressed and secreted protein (RANTES), monocyte chemoattractant protein-1, and interleukin-8 [122]. COX-2 also produces ROS [123]. The nitric oxide is generated by iNOS enzyme using arginine, oxygen, and NADPH [124]. Nitric oxide acts by various means. It can induce COX-2 enzyme [125]. 

## 9. Concluding Remarks 

Multiple sclerosis is an autoimmune disease characterized by recurrent episodes of demyelination and axonal injury mediated primarily by CD4-positive T-helper cells with a proinflammatory Th1 phenotype, macrophages, and soluble mediators of inflammation. Activated T cells are able to penetrate the blood-brain barrier, and they are then re-exposed in the CNS to their respective antigen. This in turn initiates both a cellular and humoral immune attack, finally leading to demyelination and axonal loss. 

There is significant evidence that the pathogenesis of multiple sclerosis may involve the generation of reactive oxygen species or reactive nitrogen species associated with mitochondrial dysfunction. In the initial phase of lesion formation, ROS are known to mediate the transendothelial migration of monocytes and induce a dysfunction in the blood-brain barrier. Infiltrated monocyte-derived macrophages which form the major cell type in perivascular infiltrates produce a variety of inflammatory mediators like ROS, nitric oxide, and proinflammatory cytokines, which all contribute to neuroinflammation, demyelination, axonal damage, and disease progression. The ROS enhance both monocyte adhesion and migration across brain endothelial cells. Thus, ROS are generally thought to be derived from activated inflammatory cells and to play a role in demyelination and axonal damage in multiple sclerosis. Furthermore, free radicals can activate certain transcription factors, such as nuclear transcription factor-kappa B, which upregulate the expression of many genes such as tumor necrosis factor-*α*, inducible nitric oxide synthase, intracellular adhesion molecule 1, and vascular-cell adhesion molecule 1. Also, redox reactions are involved in the activity of matrix metalloproteinases, which are important to T-cell trafficking into the CNS. 

## Figures and Tables

**Table 1:** Therapeutic strategies for multiple sclerosis.

Immunomodulator agent	Therapeutic action
Aire protein	Modulates the expression of tissue-specific antigens [33, 34].
Interferon-*β*	INF-*β* inhibits proinflammatory cytokines (TNF*α*, IL-6, IL-*β*, and IL-17) and T-cell proliferation and clinically reduces the annual rate of relapsing [64, 65].
CD28	Binds to B7.1 (CD80) and B7.2 (CD86). CD4(+)CD28null T cells+ have been found in peripheral blood of patients with MS. These cells produce high levels of IFN-*γ* and are less susceptible to regulatory mechanisms [44–46].
Inducible costimulator protein	Binds to B7H and modulates Th1/Th2 cytokine production [44–46].
CD40	CD40 is involved in the induction of IL-6 and subsequent IL-17 production [47].
PD-L1	Regulator of T-cell response [44–46].
CD58 mRNA	Increases the expression of Foxp3 transcriptional factor in Treg cells [48].
Foxp3	Regulator gene in Treg cells and determines its role in the maintenance of homeostasis and self-tolerance [39].
CD39 and CD73	Potent molecules involved in regulating IL-17 toxicity suppression [28, 49].
T cells expressing *γδ* phenotype	Provides a first line of defense against infections, through the secretion of IFN-*γ*. Clonally expanded *γδ* T cells were found in acute MS brain lesions [4].
*γδ* cells	Source of cytokine in innate immunity and exhibit a potential influence on adaptive immune responses. In models of EAE have both protective and pathogenic roles [4].
TGF-*β*	It has suppressive effects on both T- and B-cell-related immunity. The selective suppressive effects of TGF-*β*1 on proinflammatory cytokines such as IFN-y, TNF-*α*, and LT support a potential role for drugs that upregulate TGF-*β* in diseases with prominent Th1 immune response [58–62].
IL-17	Potent proinflammatory cytokine. IL-17 induces the activation of enzyme matrix metalloproteinase-3 and recruits neutrophils to the site of inflammation [63].
Paraoxonase	Enzyme able to hydrolyze preformed oxidized lipids. Exerts a protective role against oxidative damage of cells and lipoproteins [66].
Dietary polyunsaturated fatty acids	Decrease the serum levels of TNF*α*, IL-1*β*, IL-6, and nitric oxide catabolites of patients [67].
Uric acid, *α*-tocopherol, ascorbate, carotenoids, and glutathione	Free radical scavengers [68].
Transcription factor nuclear factor-E2-related factor (Nrf2)	Binds the antioxidant response element in various promoter regions, which increases the transcription of a variety of cytoprotective genes [69].
NF*κ*B	Upregulates proinflammatory gene expression [70].
Glutathione peroxidase	Antioxidant enzyme that scavenges hydrogen peroxide in the presence of reduced glutathione. Inhibits cytokine-induced NF*κ*B activation [71, 72].
